# An Evolutionarily Conserved Network Mediates Development of the *zona limitans intrathalamica,* a Sonic Hedgehog-Secreting Caudal Forebrain Signaling Center

**DOI:** 10.3390/jdb4040031

**Published:** 2016-10-20

**Authors:** Elena Sena, Kerstin Feistel, Béatrice C. Durand

**Affiliations:** 1 Institut Curie, Université Paris Sud, INSERM U1021, CNRS UMR3347, Centre Universitaire, Bâtiment 110, F-91405 Orsay Cedex, France; elena.sena@curie.fr; 2 Institute of Zoology, University of Hohenheim, Garbenstr. 30, 70593 Stuttgart, Germany; k.feistel@uni-hohenheim.de

**Keywords:** developmental biology, embryogenesis, neural, segregation, compartment, sonic hedgehog, forebrain, thalamus, holoprosencephaly, patterning

## Abstract

Recent studies revealed new insights into the development of a unique caudal forebrain-signaling center: the *zona limitans intrathalamica* (*zli*). The *zli* is the last brain signaling center to form and the first forebrain compartment to be established. It is the only part of the dorsal neural tube expressing the morphogen Sonic Hedgehog (Shh) whose activity participates in the survival, growth and patterning of neuronal progenitor subpopulations within the thalamic complex. Here, we review the gene regulatory network of transcription factors and *cis-*regulatory elements that underlies formation of a *shh*-expressing delimitated domain in the anterior brain. We discuss evidence that this network predates the origin of chordates. We highlight the contribution of Shh, Wnt and Notch signaling to *zli* development and discuss implications for the fact that the morphogen Shh relies on primary cilia for signal transduction. The network that underlies *zli* development also contributes to thalamus induction, and to its patterning once the *zli* has been set up. We present an overview of the brain malformations possibly associated with developmental defects in this gene regulatory network (GRN).

## 1. Introduction

In the last decades, functional experiments in numerous model systems provided crucial insights into the early molecular, cellular, and morphological events underlying forebrain development. They revealed the importance of brain signaling centers: small groups of specialized cells acting as local sources of secreted factors. These secreted factors regulate survival, proliferation, and early patterning of neuroepithelial cells and facilitate compartmentalization of the neuroepithelium into functional histological units [1,2,3,4,5,6,7].

The forebrain (also called prosencephalon) is derived from the most anterior part of the neuroepithelium, the prosencephalic neural plate. Its rostral part, the telencephalon, develops into the cerebrum, which gets divided into the cerebral hemispheres. The caudal forebrain or diencephalon is situated between the cerebral cortex and the midbrain. It gives rise to the thalamic complex, a bilateral structure that contains the pre-thalamus (ventral thalamus) rostrally and the thalamus (dorsal thalamus) caudally. 

Development of the thalamus is orchestrated by a brain signaling center called the *zona limitans intrathalamica* or *interthalamica* (*zli*), also referred to as the mid-diencephalic organizer (MDO) [3]. The *zli* emerges after the completion of neural tube closure and is characterized by a dorso-ventrally (DV) extending gene expression domain of the morphogen Sonic hedgehog (Shh). Shh secreted from the *zli* and the basal plate participates in the survival, growth, and patterning of neuronal progenitor subpopulations within the thalamic complex [8,9,10,11,12,13,14,15].

In-depth reviews detail the role of Shh in diencephalic patterning [3,13,16,17]. Here we focus on the principles underlying emergence of the *zli*. Specifically, we describe the network of transcription factors and *cis-*regulatory sequences that confer competence for *zli* establishment. We review work analyzing their evolutionary conservation. We also highlight the contribution of signaling pathways to *zli* formation, discuss implications for the fact that Shh relies on primary cilia for signal transduction and speculate about *zli* development for the etiology of brain malformations.

## 2. The Zli: A Unique Shh-Expressing Compartment in the Caudal Forebrain

### 2.1. Forebrain Morphology and Zli Positioning

**Figure 1:**
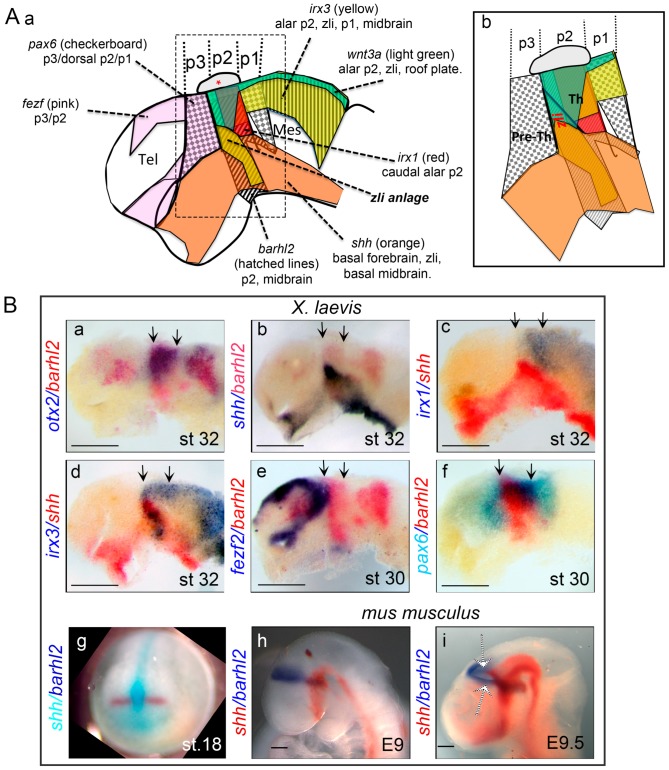
Schematic of *Xenopus* (*X.*) *laevis* forebrain markers and flat-mounted neural tubes from *X. laevis* and *Mus musculus* embryo after whole-mount double *in situ*-hybridization (WM-dISH). (**A**) Schematic of *X. laevis* forebrain markers at st. 30. (**a**) Areas of expression are shown and indicated for *fezf2* (pink), *pax6* (checkerboard pattern), *irx3* (yellow), *wnt3a* (light green), *shh* (orange), *barhl2* (hatched lines), and *irx1* (red). (**b**) Enlargement of the diencephalic part indicated by a rectangle in (**a**). The pineal gland located on top of p2 is marked by an asterisk. The future *zli* is indicated in (**b**). p: prosomere; Tel: telencephalon; Mes: mesencephalon; Pre-Th: pre-thalamus; *zli: zona limitans intrathalamica*; Th: thalamus. (**B**) WM-dISH is performed using *otx2*, *irx1*, *irx3*, *fezf2*, *pax6,* and *shh* or *barhl2* as probes on (**a**–**g**). *X. laevis* and mouse embryos at different developmental stages as indicated. Neural tubes (**a**–**f**) or heads (**h**,**i**) of representative embryos, dissected and flat-mounted, are shown from a side view, dorsal up, anterior left except for (**g**), shown in anterior view, dorsal side up. The markers and stages are indicated. Black arrows indicate the rostral and caudal boundaries of p2. White arrows indicate the bilateral *zli*. Scale bar: 0.5 mm.

On the basis of morphology [18,19,20,21] and gene expression [22,23], the diencephalic primordium is divided into three transverse segments called prosomeres (p) that generate three distinct histogenic fields: p3, or anterior parencephalon, corresponds to the presumptive prethalamus and the eminentia thalami; p2, or posterior parencephalon, gives rise to the epithalamus and the thalamus; and p1, or synencephalon, generates the presumptive pretectum (reviewed in [17,24,25,26,27]). Each prosomere is divided into a ventral (basal) and a dorsal (alar) part (Figure 1A).

The *zli* demarcates the boundary between pre-thalamus and thalamus, separating the posterior diencephalon (p1 and p2) from the anterior diencephalon (p3) (Figure 1; [28,29,30]). This position corresponds approximately to the junction between the prechordal neuraxis overlying the prechordal mesendoderm, and the epichordal neuraxis overlying the notochord [9]. The *zli* is defined by a dorsalward continuation of *shh* expression into the alar plate of the diencephalon (Figure 1A,Bb–d). The *zli* is the last brain signaling center to emerge: its dorsal progression within the alar plate starts at stage 24 in frog [12]), HH12 to HH26 in chicken [28,29,31], E9 in mouse, and between the 12- and 15-somite stage in zebrafish [32]. It is unique in brain regionalization because it represents the only neural area in which Shh, normally a DV patterning signal, regulates anterior-posterior (AP) regionalization [33,34].

### 2.2. Shh Expression Initiates and Demarcates Zli Development

During gastrulation and early neurulation, *shh* is expressed in the prechordal plate and notochord. Notochord-derived Shh activates the Hedgehog signaling pathway and its own neural midline expression in the overlying neuroepithelial cells ([35,36]; reviewed in [37]). In the posterior part of the neural tube, *shh* gene expression remains restricted to the medial floor plate, whereas in the mesencephalon and prosencephalon *shh* expression spreads from the floor plate to the basal plate. It is only at the p2 to p3 boundary that a triangle-shaped expression of *shh* extends dorsalward (i.e., more lateral) on either side of the diencephalic walls (Figure 1; reviewed in [37]). Studies in chicken revealed that a sequential induction process, initiated by Shh secreted by floor plate cells, underlies the appearance of the *shh*-expressing line of cells corresponding to the *zli* [38]. This homeotic induction of *shh* occuring from one cell to its neighbor is not understood. It could necessitate specific Shh maturation and secretion processes, together with modification at the level of the primary cilium that mediates Shh read-out. Because the *zli* is not formed through cell migration from the basal to the alar plate, the *zli* is considered an alar structure [38]. Observations in frog, chicken, and mouse demonstrate that a continuous source of Shh signals, provided *in vivo* by secretion of Shh initially from floor plate cells, and then from the basal plate, is strictly necessary both for induction of *shh* expression within the *zli* and for the correct segregation of *zli* cells from the thalamus [12,28,32,38,39]. In zebrafish, however, a small domain of *shh* expression at the position of the *zli* appears in *smoothened* (*smo*) mutants, in which signaling downstream of all Hedgehog family ligands is defective [40,41]. Similarly, a population of “*zli* cells” develops in a quarter of zebrafish *cyclops* mutants, which lack a floor plate in the diencephalon [32,38,39,42]. Taken together, these observations suggest that, contrary to observations in other model organisms, the continuous presence of a neural midline-derived Shh signal is not required for emergence of the *zli* in zebrafish.

### 2.3. Physical Separation: The Zli as a Tissue Compartment

To fulfill their localized patterning function, cells of a signaling center need to be arranged in a coherent compartment with clear boundaries and lineage restriction. This enables the signaling center to maintain its position relative to the surrounding tissue [24,28,29,43] (reviewed in [17,44]). An analysis of chick diencephalon development revealed that early on, the caudal forebrain territory is not overtly segmented [29]. Development of the *zli*, however, correlates with acquisition of cell lineage-specific properties. Cell movements become restricted at both the anterior and posterior limits of the *zli* and *zli* formation is associated with acquisition of properties observed at tissue boundaries: 1) interkinetic nuclear migration movements are disrupted and 2) markers of immiscible interfaces such as chondroitin sulfate proteoglycans, laminin, weakly polysialylated neural cell adhesion molecules, and vimentin are detected within the *zli* territory [28,29]. These studies established that the *zli* is a narrow compartment with cell lineage-restricted boundaries, introducing physical separation of the diencephalon into the prethalamus anteriorly and the thalamus posteriorly [24,28,29,31].

This physical separation brought about by lineage restriction within the *zli* may in addition be instructive for AP segmentation on a forebrain-wide scale. According to the prosomeric model the forebrain becomes subdivided into an anterior and a posterior part. Anteriorly, the so-called secondary prosencephalon contains the telencephalon, optic vesicles, and hypothalamus. More posteriorly, the alar plate of the diencephalon is divided into the anterior p3, p2 and p1 ([28]; reviewed in [17,25]). Note that whereas the prosomeric model differentiates p2 and p3 basal plates from the anterior basal hypothalamus, in *zebrafish* the hypothalamus is thought to develop within the basal diencephalon, a difference that may generate misinterpretations. Partitioning of the anterior forebrain from the remainder of the neural tube partly relies on the distinct processes inducing specification of the prechordal versus epichordal neural plate. It is still under debate whether the alar plate of p3, which generates the prethalamus, is under the influence of the chordal, or of the prechordal mesendoderm [25,29]. However, cell lineage analysis indicates that cells do not segregate between the prethalamus and the secondary prosencephalon [29]. Moreover, the competence of the prethalamus (p3) to respond to instructive factors such as fibroblast growth factor (Fgf) 8 is different from that of the other diencephalic prosomeres (p2 and p1) [45,46]. This raises the question whether the *zli* could represent a pivotal structure along the neuraxis segregating a “large prosencephalon” from the remainder of the neural tube. At minimum, the *zli* separates territories with differential competence to respond to morphogens [29,47].

In conclusion, the *zli* is a narrow compartment, located in the alar plate of the caudal forebrain that expresses *shh*. It is the last signaling center to form. It introduces segmentation within the diencephalon and has an important function in diencephalic patterning. The *zli* is characterized by the same positioning as similar gene expression patterns in all model organisms. However, the mode of its formation may vary between species. From studies in zebrafish, frog, chicken and mouse, it is now possible to propose a model for the GRN and the inductive cues allowing formation of this unique signaling center.

## 3. A Role for Wnt Ligands in Generating a *Zli*-Permissive Compartment

**Figure 2:**
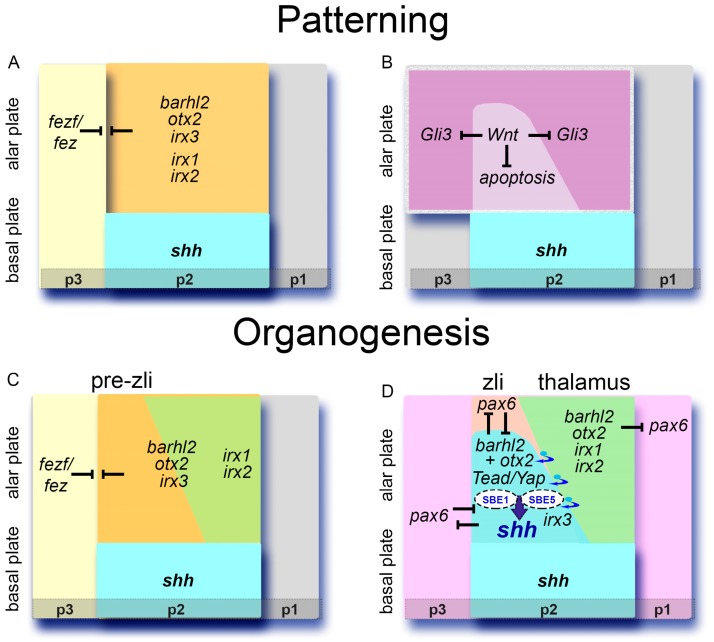
A model for *zli* formation. (**A**) During neural plate patterning the diencephalic primordium expresses *otx2, barhl2* and *irx1, irx2, irx3* (orange). Cross-repressive interactions between Fezf/Fez and Irx homeoproteins contribute to formation of the p2/p3 alar border. *shh* (light blue) is expressed in the p2 basal plate. (**B**) Concomitantly, a Wnt-mediated signal promotes survival of *zli* anlage cells, and *gli3* expression is repressed through a Wnt-mediated signal in a narrow band of cells that correspond to the *zli* primordium. (**C**) At the onset of *zli* formation a permissive environment is established in the future *zli,* and *zli* cells acquire the competence to express *shh*. The *zli* anlage is characterized by the expression of *barhl2, otx2, irx3* (orange), and the future thalamus by the expression of *barhl2, otx2, irx1/2/3* (green). At the onset of *zli* development, *irx3* is expressed in the future thalamus territory. (**D**) Using a sequential induction mechanism, Shh secreted by the basal plate initiates the spreading of *shh* expression into the pre-*zli* domain. Transcription factors (TFs) including Otx1, Otx2, Barhl2, Yap–Tead, and possibly unidentified TFs (TF-X) are recruited to the enhancers SBE1 and SBE5 to initiate *shh* transcription within the *zli*. Under the inductive influence of secreted N-Shh, some *irx3-*expressing cells located in caudal p2 sort out from thalamic cells. Shh signaling from the *zli* induces the repression of *pax6* within the mid-diencephalic furrow. Pax6 together with unidentified dorsal signals could in turn prevent *Shh* from being expressed beyond the *zli*. The thalamus (p2) and the prethalamus (p3) receive a combination of Shh signals coming from two orthogonally oriented signaling centers, the *zli* and the basal plate. Together, they establish a morphogenetic gradient of Shh protein that patterns the thalamus posterior to the *zli* and the prethalamus anterior to the *zli*. p: prosomere; *zli*: *zona limitans intrathalamica*.

Observations in zebrafish, amphibian, and chicken have highlighted early and late roles for Wnt ligands in *zli* positioning, induction, and development [48,49] (Figure 2). Canonical Wnt signaling is involved in AP patterning of the forebrain [50] (reviewed in [51]). Receptors, ligands and modifiers of the Wnt pathway are expressed during caudal forebrain regionalization. Specifically, expression of the Wnt ligands Wnt3, Wnt3a, and Wnt8b mark the alar plate of p2 and the *zli* primordium [12,31,49].

In zebrafish, lack of canonical Wnt signaling brought about by a Wnt signaling antagonist induces loss of the pre-*zli* territory. Conversely, enhancement of Wnt signaling using the Glycogen Synthase Kinase 3β (GSK3β) inhibitor BIO leads to a broader expression domain of *shh* at the *zli* [48]. The depletion of both Wnt3 and Wnt3a leads to an increase of apoptosis and a loss of the diencephalic organizer primordium indicating that Wnt3 and Wnt3a are normally required for survival of *zli* anlage cells. Interestingly, the effect of canonical Wnt signaling on the survival of *zli* anlage cells is restricted to a time window of 4 hours during somitogenesis. In embryos depleted for Wnt3 and Wnt3a the size of the prethalamic and thalamic markers *fezf2*, *irx1b* and *otx2* expression domains are unaltered (Figure 1A and Figure 2). In Wnt3/3a depleted embryos the concomitant depletion of *fezf2*, or of Irx1b, activity rescues *zli* formation, indicating that both Fezf2 and Irx1b normally restrict the *zli* territory [48]. Therefore, Wnt3/3a function is required for maintenance of the *zli* anlage, but not for the maintenance of the prethalamus and thalamus territories (Figure 2).

A study by Martinez-Ferre et al. reveals a requirement for a Wnt8b-mediated signal as a permissive step for the subsequent induction of *shh* expression and for emergence of the *zli* in the diencephalic primordium [49]. Gli3 is a Shh-regulated transcriptional repressor [52,53,54]. During early patterning stages of the neural plate *gli3* transcripts are detected within the alar neural plate and specifically in the alar diencephalon. In chick, this wide expression domain gets restricted to a narrow band of cells at the center of the *wnt8b* expression domain. The transverse alar stripe of *wnt8b* which is now devoid of *gli3* expression is the prospective *zli* anlage [11]. Over-expression of *gli3* in the *zli* inhibits *shh* induction, indicating that the local repression of *gli3* is necessary to allow *shh* homeotic induction during *zli* formation. Martinez-Ferre et al. demonstrated that the local downregulation of *gli3* at the future *zli* requires a Wnt-signal, which in chicken is mediated by the presence of Wnt8b. Wnt8b signal appears necessary to locally downregulate *gli3* at the center of the *wnt8b* expression domain. It is, however, not sufficient, as the entire domain that expresses *wnt8b* does not lose *gli3* expression. Finally, once Shh is expressed in the *zli,* inhibition of the Wnt pathway does not have any effect on its maintenance [49]. 

In conclusion, while Wnt-mediated signals are important for maintenance of the *zli* anlage, and to generate the permissive conditions for the activation of Shh in the *zli*, they must act in collaboration with a combination of factors at the prethalamic (Fezf/Fez) and thalamic (Barhl2, Irx3, and Otx2) forebrain borders.

## 4. Laying the Ground for the *zli*: Otx2 and Barhl2 Binding to *cis-*Regulatory Sequences Confers Competence for *zli* Formation

During neural induction, an underlying pre-pattern, partly encoded by TFs, emerges in the neural plate. These early patterning cues contribute to the specification of forebrain territories and influence the way in which neighboring cell populations respond differentially to similar morphogens [46,55,56]. Analysis of TF expression dynamics in the anterior neural plate provides crucial information about the cues involved in emergence of the *zli* and reveals that *zli* induction starts during neurulation. 

Two TFs are especially involved in conferring competence to the future *zli* tissue to express *shh*. Orthodenticle homeobox (Otx) 1 and 2 are homeodomain-containing proteins involved in specification and regionalization of the forebrain (reviewed in [57,58]). The *otx2* expression territory marks the anterior neural plate from gastrulation onwards [59]. In both zebrafish and frog *otx* expression decreases in the telencephalic territory during neurulation. At the onset of *zli* development *otx* expression is restricted to the p2 and midbrain territories ([12,32]; Figure 1Ba). The Bar-class homeodomain-containing (BarH) Barh1 and Barh2 are also homeodomain-containing TFs (reviewed in [60,61,62]). Transcripts encoding BarH-like (*barhl) 2* are detected in the diencephalic primordium of amphibian [63,64], zebrafish [65], and mouse [66,67]. Similar to *otx* genes, at the onset of *zli* development *barhl1* and *barhl2* expression are restricted to prosomere p2. While *barhl2* is expressed in the entire p2, *barhl1* expression is restricted to basal p2 ([68,69]; Figure 1A,Bb).

In zebrafish, the lack of Otx1l/2 (the zebrafish ortholog of Otx2) function leads to absence of the *zli* and subsequently of *zli*-dependent target genes. With a lack of Otx function, the thalamus is mis-specified prior to, and independently from, *zli* formation. The adjacent territories of the prethalamus and pretectum expand into the mis-specified territory and form a new interface. Mouse embryos with reduced *otx1/2* transcripts similarly show a lack of *shh zli* expression [67]. Therefore, the presence of Otx1/2 is required to establish a competence area, allowing induction of *shh* in the *zli* [32,67,70]. In Otx-depleted zebrafish embryos, ectopic expression of Otx rescues formation of the *zli* solely within the diencephalic territory, anterior to the presumptive thalamus [32]. In *X. laevis* embryos depleted for Barhl2, development of the *zli* is abolished [12]. Similarly, in *barhl2*^−/−^ mice, *shh* expression within the *zli* is significantly reduced [67,71]. Barhl2-depleted *X. laevis* embryos resemble zebrafish Otx1l/2-depleted embryos: although most forebrain markers are unaltered, the embryos exhibit defects in *shh* expression in the *zli* and in the formation of the mid-diencephalic furrow [12,71]. Noteworthy, in zebrafish loss of Otx2 induces a loss of *barhl2* expression while *otx2* expression is maintained in Barhl2-depleted *X. laevis*. Therefore, Otx1l/2 proteins are necessary for maintenance of *barhl2* in the future *zli* territory, and the loss of *barhl2* contributes to the *zli* defects observed in Otx-deficient zebrafish [12,32]. 

Synergistic activities of Barhl2 and Otx2 in *zli* formation are confirmed by analysis of the *cis-*regulatory-motifs controlling *shh* expression within the mouse *zli*. Yao et al. identified two enhancers, SBE1 and SBE5, driving *shh* expression within the *zli*. Both enhancers function in a partially redundant manner and their activity relies on six position-independent motifs directly regulated by a combination of Otx2, Barhl2 and the TEA-domain family member 2 (Tead2), a key mediator of Hippo signaling, and its co-transcriptional activation partner Yap. Using large-scale genomic approaches associated with bioinformatic analytical tools, Yao et al. characterized a 116-bp homology block, referred to as SBE1-like-enhancer, present in enhancers scattered throughout the mouse and human genomes. The SBE1-like-enhancer sequence is conserved from human to zebrafish. Using luciferase reporter assays, chromatin immunoprecipitation (ChIP), and transgenic mouse reporter assays, Yao et al. showed that the six motifs are necessary and sufficient for full enhancer activity and paired three motifs with cognate transcription factors. Motifs 1 and 6 correspond to recognition sequences for Otx1/Otx2 and Barhl2, respectively. Indeed, the combined action of Barhl2 and Otx2 resulted in a synergistic induction of reporters whose activity is under the control of SBE1-like enhancers containing motifs 1 and 6. Similarly, Tead2 and Yap recognize and contribute to *shh* expression through binding on motif 2. Embryos with conditional loss of Yap1 showed a selective reduction in *zli shh* expression. Tead2 and Yap activities on *zli* formation have not been assessed in species others than mouse. The second enhancer SBE5 contains a cluster of permuted motifs similar to those identified in the SBE1 enhancer in the absence of any other overt sequence homology. This second enhancer, located in the vicinity of the *shh* locus, performs equivalently to SBE1 in cell-based reporter and ChIP assays. Deletion of both enhancers in mouse entirely abolishes expression of *shh* within the *zli* [67].

Taken together, these studies reveal the presence of a “*zli* developmental cassette” that uses two main TFs, Otx2 and Barhl2, in combination, at least in mice, with the Tead2-Yap1 activation complex and conserved *cis-*regulatory motifs to induce *shh* expression in a narrow band of cells within the anterior brain.

## 5. Hedgehog Sequential Induction Process in *zli* Formation: Shh Is a Secreted Signal Read out through a Primary Cilium

During *zli* formation the N-terminal part of Shh secreted by basal plate cells is released into the ventricular lumen and N-Shh activates its own expression only in its neighboring cell [38]. Pre-*zli* cells respond differently to Shh compared to neighboring prethalamus and thalamus cells. The cues controlling sequential induction and the differential response to Shh are not fully deciphered. Modifications in Shh maturation and secretion, and/or in the primary cilium that mediates Shh read-out, could participate in this unique process.

### 5.1. Shh Maturation and Secretion

The secretion of Hedgehog (HH) proteins into the ventricular lumen necessitates that HH proteins undergo an autocatalytic internal cleavage associated with the addition of lipid molecules, specifically cholesterol and palmitic acid moieties (reviewed in [72]). Cleavage confers a hydrophobic character to HH that is required for its association with the cell membrane. HH proteins truncated at the site of internal cleavage diffused more largely [73]. The internal cleavage occurs in the endoplasmic reticulum and produces a 20 kD amino-terminal domain (N-HH) and a 25 kD carboxy-terminal domain (C-HH) [74,75,76]. The C-HH part recruits a cholesterol molecule that binds to the C-terminus of N-HH [76,77]. The binding of cholesterol to N-HH is essential in limiting the range of HH signaling. In mouse, N-HH lacking cholesterol has an extended signaling range in the limb bud [78]. Besides cholesterol, the N-HH protein is also modified by the attachment of a palmitic acid group on its N-terminal part that has proved important for HH activity [79]. The transfer is mediated by an acyltransferase named Skinny Hedgehog (SKI) in *Drosophila* and mouse and Hedgehog acyltransferase (HHAT) in humans [80,81,82]. Mouse embryos lacking N-HH palmitoylation exhibit decreased Shh signaling in limb buds [83] as well as in ventral forebrain formation [84].

Different mechanisms have been described for the release of processed N-HH (pN-HH) into the extracellular space. pN-HH is strongly hydrophobic. It can be secreted as a monomer but needs to bind to other secreted proteins. In *Drosophila*, the transmembrane protein Dispatched-1 (Disp1) [85] binds to pN-HH along with the secreted protein Scube2. Disp1 and Scube2 bind to different parts of the cholesterol attached to N-HH and promote the release of pN-HH from the cell surface [86,87]. There is also evidence that pN-HH secretion is mediated through a lipoprotein complex [88,89]. Lipoproteins consist of a phospholipid monolayer that embraces the lipids present in the pN-HH leaving the hydrophilic areas outside, decreasing the strong hydrophobicity of the pN-HH molecule. In *Caenorhabditis elegans* (*C. elegans*) [90] and *Drosophila* [91], an exosome-mediated release has been described. In vertebrates pN-HH can also be released in a vesicular form [92].

### 5.2. The Shh Signal Is Read through a Primary Cilium 

Shh-related patterning defects in mice carrying mutations in genes essential for cilia function revealed that Shh signaling in vertebrates requires the presence of primary cilia [93]. Cilia are small, membrane-sheathed cell protrusions that occur on almost all cells during development and adulthood. Motile cilia can be bent by dynein motors, which results in rotational or beating movement, whereas primary cilia are immotile. Owing to the fact that their membranes are studded with a range of different mechano- and chemo-receptors, primary cilia are considered highly specialized sensory organelles [94]. Signal transduction from the sensory primary cilium requires motor protein-driven transport along axonemal microtubules, termed intraflagellar transport (IFT). IFT is required for Hedgehog signal transduction since Patched (Ptc), a twelve-transmembrane domain receptor, Smo, a G-protein coupled receptor, and the zinc finger containing Gli family of TFs are moving into, within, and out of the cilium, depending on pathway activation status ([95]; reviewed in [72,96,97,98]).

**Figure 3:**
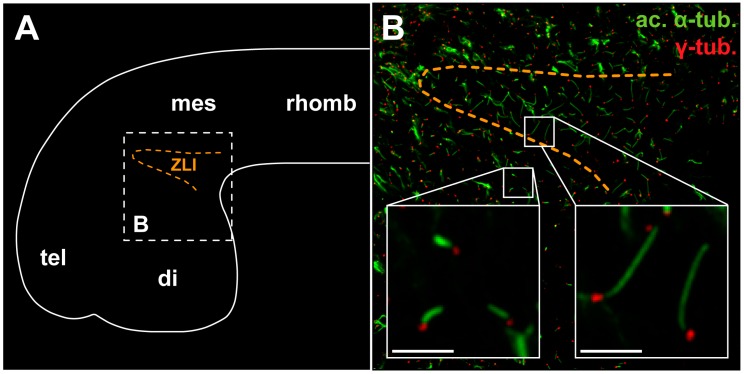
Elongated cilia on the *zli.* (**A**) Schematic overview of the *Xenopus* tadpole brain comprised of (from anterior to posterior) telencephalon (tel), diencephalon (di), mesencephalon (mes) and rhombencephalon (rhomb). The position of the *zli* is indicated by orange dashed line. (**B**) Magnification of area indicated by white dashed line in (**A**). Whole mount immunofluorescence staining on the ventricular surface of a tadpole brain. Antibodies detecting acetylated alpha-tubulin (ac. α-tub.) and gamma-tubulin (γ-tub.) mark cilia axonemes and basal bodies, respectively. Note that *zli* cilia are markedly elongated compared to cilia on neighboring cells (scale bars: 5 µm).

During embryogenesis, neural tube cells in mouse [93,99], chicken [100], *X. laevis* [101], and zebrafish [102] carry primary cilia on their apical surface. These are crucial for Shh signal transduction governing DV patterning of the neural tube [93]. Cilia have also been identified on ventricle-contacting cells specifically in the forebrain region in mouse [103] and on diencephalic cells corresponding to the region of the *zli* in *X. laevis* (Figure 3; [104]). Interestingly, cilia on cells within *shh*-expressing brain signaling centers including the floor plate, rhombomere boundaries, and *zli* are specifically elongated compared to surrounding primary cilia on non-*shh*-expressing cells ([100,101,104] and Figure 3). This could indicate a way of differentially adapting signaling pathway activation, as longer cilia seem to correlate with reduced pathway activation [100].

In conclusion, the mechanisms controlling Shh secretion and diffusion rate in the context of *zli* development are still being investigated. The strong hydrophobic character of N-Shh suggests that it diffuses very slowly and tends to stay close to the membrane surface. This could at least partly explain the sequential induction process observed during *zli* development. Moreover, a primary cilium is present in diencephalic progenitor cells. The primary cilium is structurally different on pre-*zli* cells versus prethalamic and thalamic cells and little is known on the biological consequences of such differences.

## 6. Building Compartments: The Iroquois Genes Refine the *Zli* Borders

The Iroquois (*irx*) genes encode for homeodomain-containing TFs, highly conserved from *Drosophila* to mammals (reviewed in [105,106]). Most vertebrates contain six *irx* genes grouped in two paralog clusters of three genes each [107,108]. The *irxA* cluster contains *irx1*, *irx2* and *irx4*, while the *irxB* cluster corresponds to *irx3*, *irx5* and *irx6*, and *irx7* in zebrafish. The Irx proteins participate in defining territories and in specifying cell identity. *irx* gene activities in boundary formation have been previously described (reviewed in [105]).

Observations in zebrafish and amphibian promote the idea that Irx factors participate in acquisition of *zli* compartment identity [12,32]. In all species studied, *irx1*, *irx2*, and *irx3* are co-expressed in the anterior neural plate during the early stages of neural patterning and mark the future p2 (Figure 1 and Figure 3; [63,109,110]). In zebrafish, *irx3b* is strongly expressed in the developing *zli* together with *irx5a*, *irx5b* and *irx7.* The *irx3b/5/7* expression pattern subdivides the *zli* into distinct DV domains. In contrast, *irx1a*, *irx1b*, *and irx2a* expression domains abut the *zli* posteriorly [110]. In zebrafish, *irx1b* function is dispensable for *zli* development; however, depletion of the *irx1* orthologs *irx1b and irx7* induces a posterior shift of the *zli* caudal border and an expansion of the *shh* expression domain at the expense of the thalamic field [32,70].

Expression analysis of *irx* genes during *X. laevis zli* development reveals a temporally dynamic process [12]. At neural plate stages, *irx1*, *2* and *3* are co-expressed in the future p2 domain expressing *barhl2* and *otx2*. At the onset of *zli* formation the rostral p2, i.e., the future *zli*, expresses solely *irx3*, whereas the caudal p2, i.e., future thalamus, expresses *irx1*, *2* and *3*. After the *zli* has fully developed, the rostral p2 expresses *irx3* and *shh*, and the caudal p2 expresses *irx1* and *irx2* (Figure 1A,Bc,d and Figure 2 and [12]). Furthermore, the mis-expression of the Irx factors within the *barhl2*/*otx2*-expressing field in frog indicates that the ratio of Irx3 to Irx1/2 is essential for *zli* specification and for establishment of the *zli* posterior boundary. Both overexpression of *irx3* as well as *irx1*/*2* depletion promotes the acquisition of a *zli* fate at the expense of a thalamic fate. It is unknown whether Irx1, Irx2, and Irx3 proteins regulate one another’s expression.

Observations in chick embryos are divergent from those described in other vertebrates. Chicken *irx3* is described as being expressed posterior to the *zli* and appears to have a repressive function on *zli* formation [10]. In chicken, Irx3 does not appear to participate in *zli* development but in establishment of the differential cellular competence to respond to Shh signaling from the *zli* [46]. Misexpression experiments in the caudal forebrain through in ovo electroporation approaches indicate that Irx3 together with Pax6 alters the competence of caudal forebrain cells to respond to both Fgf8 and Shh. Irx3 ectopic expression in the prethalamic anlage induces expression of thalamic markers and represses prethalamic markers [46].

In conclusion, numerous observations indicate that Irx activities are important to establish the anterior and posterior borders of the *zli*, and to acquire cell segregation properties. Irx activities may vary depending on species. Whether the Irx proteins are strictly required for the expression of *shh* within the *zli* territory, and what is their exact function in *zli* formation, remain open questions.

## 7. Sticking Together: GRNs and Signaling Pathways Modulate *Zli* Cell Differential Adhesive Properties and Proliferation Rate

Compartments exhibit specific features: they are characterized by a reduced rate of cell proliferation and cells of neighboring compartments separate along boundaries. Cells segregate from one another based on differences in the adhesive properties (affinity) of their cell surfaces. At the boundary, cells deposit an extracellular matrix that acts as a mechanical barrier between different cell populations. Establishment of adhesive differences constitutes the first step of lineage restriction, whereas fence-type mechanisms might stabilize compartments at later stages (reviewed in [44,111]).

### 7.1. Canonical Wnt Signaling Regulates Thalamic Cell Adhesiveness and Segregation

Various canonical Wnt ligands are markers of the alar plate of p2. Analysis of caudal forebrain proliferation kinetics in chicken and mouse reveal that *zli* cells divide slowly relative to cells in their flanking territories [43,112]. β-catenin, the main effector of the canonical Wnt pathway, controls neuroepithelial cell proliferation (reviewed in [113,114,115]). β-catenin mediates the interactions between the intracellular cytoskeleton and the cadherins, a group of cell-cell adhesion proteins important in the formation of neural boundaries. Members of the cadherin superfamily mediate differential adhesive properties and are expressed differentially in the forebrain subdivisions. Therefore, it is thought that cadherins participate in formation of compartment boundaries (reviewed in [116,117,118]). In the zebrafish thalamus, β-catenin regulates the expression of protocadherin 10b (Pcdh10b, formerly known as OL-protocadherin; [118]). Alteration of *pcdh10b* expression in the thalamus territory leads to an intermingling of thalamic cells with the neighboring brain areas, predominantly with the pretectum, indicating an important role for *pcdh10b* in thalamus separation. Stabilization and nuclear translocation of β-catenin leads to a broadening of the expression domain of *pcdh10b,* whereas inhibition of Wnt signaling decreases *pcdh10b* expression. Therefore, the Wnt canonical pathway participates in acquisition of the adhesive differences supporting differential cell segregation behaviors within p2.

### 7.2. The Notch/Delta Pathway Contributes to Correct Separation of the Zli and Thalamic Fields

Notch signaling mediates lateral inhibition in embryonic tissues and during neural development (reviewed in [119]). *delta* as well as *radical fringe (rfng)* and *lunatic fringe* (*lfng*), two glycosyltransferases that regulate Notch signaling, are expressed and have been involved in the establishment of boundaries. In zebrafish, *in vivo* activation of the Notch pathway directs cells to the rhombomere boundaries, whereas inhibition of Notch activity excludes cells from boundaries [120]. In avian embryos, a forebrain wedge-shaped domain referred to as the “pre-*zli*” is characterized by a gap in the expression of *lfng*. Ectopic expression of *lfng* in this “pre-*zli*” compartment results in sorting of the electroporated cells into the *lfng*-expressing border regions, indicating that *lfng* contributes to specification of the *zli* borders and acquisition of its compartment properties [31,121]. Of note, cell lineage analysis suggests that this “pre-*zli*” domain has compartment properties and is thought to collapse during development to form the final *zli* [31]. However, the mechanism allowing this allosteric growth are not yet explained, and are not attributable to either cellular movements in the epithelium [28] or to cell death [31].

### 7.3. The TFs Barhl2 and Irx Facilitate Acquisition of Zli Compartment Properties

As described above, Barhl2 participates in *shh* induction within the *zli* territory. Moreover, Barhl2 acts as a brake on p2 neuroepithelial cell proliferation and plays a key role in the maintenance of diencephalic neuroepithelial architecture by limiting activation of the Wnt canonical pathway [64]. In fly imaginal discs, the reduced activity of *irx* genes promotes cell proliferation by accelerating the G1 to S transition whereas their increased expression causes cell-cycle arrest contributing to the size determination of the imaginal disc [122]. Irx proteins act on establishment of distinct cell affinities in dorsal versus ventral cells in the *Drosophila* eye [123]. In zebrafish, *iro7* is required for the proper positioning of the prospective r4/r5 hindbrain rhombomeric boundary [110,124]. Taken together, these observations indicate that Barhl2 along with the Irx factors could participate in limiting *zli* cell proliferation as well as enabling adhesion and facilitating acquisition of *zli* compartment properties (reviewed in [62]).

In conclusion, the Wnt, Shh, and Notch signaling pathways act in a coordinate way to drive segregation of *zli* cells. The identification of genetic and epigenetic modifications initiated by the co-expression of *otx2, barhl2,* and *irx* genes in the presence or absence of Shh should identify the molecular cues driving segregation of *zli* and thalamic cells.

## 8. The Programming of A “*Zli*-Like” Structure in *X. laevis* Animal Cap Explants Confirms Otx2, Barhl2 and Iroquois Cell Autonomous and Non-Autonomous Activities in *Zli* Formation

In amphibian, cells from the roof of the blastocoel are pluripotent. These cells can be isolated and programmed to generate tissues through manipulation of gene expression—injection of synthetic mRNA or morpholino (MO)—or induction by secreted factors. The investigation of the GRN underlying *zli* formation was performed using such explants [12,125]. 

In the presence of Shh, explants co-expressing *barhl2*, *otx2* and *irx3* acquire a *zli-*like identity. The shape of the *shh*-expressing domain observed in the programmed explants—wide where in contact with the source of Shh signal and pointed at its other extremity—is consistent with an induction process occurring sequentially from one cell expressing *shh* to the next. Concomitant in time with acquisition of a Shh-expression program, *zli*-like cells acquire cell segregation properties that depend on the presence or absence of a Shh signal: explant cells mimic their *in vivo* segregation behaviors. Moreover, when grafted into a developing neural plate and continuously exposed to a Shh signal, neuroepithelial cells co-expressing *barhl2, otx2,* and *irx3* form an ectopic *zli in vivo* [12,125].

These approaches confirmed that the GRN which governs competence and refinement of the *zli* can be reconstructed in an *ex vivo* system, and a cell-autonomous role for Otx2 and Barhl2 [12,125]. Moreover, these data indicate that there could be an ongoing recruitment of *irx3* expressing cells from the thalamus into the *zli* during *zli* formation. It is not known whether Shh induces the segregation of *zli* and thalamus cells *in vivo,* however, such recruitment would participate in giving the *zli* its wedged shape, wide at the bottom and pointed at its extremity, a morphology observed in most vertebrate species (Figure 1Bb and Figure 2D). Finally, these data also reveal that the efficiency of *shh* induction—i.e., the average size of the *shh*-expressing area—is increased in the presence of a thalamus-like explant [12,125]. Further experiments are necessary to determine whether the thalamus territory through a cell non-autonomous mechanism facilitates the induction of *shh* and contributes to the dorsal progression of *shh* expression.

## 9. Inductive Cues and *Zli* Positioning: A Role for the p2/p3 Border and for Fezf/Fez TFs

Using grafting experiments in chicken embryos, Vieira et al. showed that an ectopic border between neural tissue from a prechordal (defined as being *six3*-positive) and epichordal (defined as being *irx3*-positive) origin is sufficient to induce an ectopic *zli* [9]. Indeed, comparison of *barhl2* and *shh* expression patterns during neurulation support the idea that the *zli* forms in immediate proximity to the interface between the prechordal neuraxis, induced by prechordal plate mesoderm, and the epichordal neuraxis, induced by the chordamesoderm ([29,30]; Figure 1Bg). In any case, the *zli* anterior border develops at the interface between the expression domains of the FEZ family zinc finger 2 (*fezf2*), which marks the alar plate of p3 rostral to the *zli*, and *irx3* that marks p2 Figure 1Be; [30,38,39,49]).

*fez-like (fezl)* genes are highly conserved during evolution from flies to men. They are expressed in the presumptive pre-thalamus during early segmentation stages but are not detected within the *zli* territory (Figure 1A,Be and Figure 2). Functional analysis of Fez and Fezl in zebrafish and mouse indicate an evolutionarily conserved role for these TFs in *zli* formation. Analysis of E12.5 mouse embryos in which both Fez and Fezl genes have been disrupted reveals defects in both prethalamus and diencephalic development, including loss of *shh* expression in the *zli*, ectopic expression of *pax6* in the mid-diencephalic furrow, and extension of *wnt3a* expression within the p2 alar plate. In contrast, in zebrafish the knock down of *fezl* results in anterior expansion of the *zli*, associated with concomitant expansion of *irx3a*. This difference in loss of function phenotypes remains unexplained. It could be species-dependent or it could be due to a difference in levels of expression. The zebrafish *fezl* morphant represents a weaker loss of function of *fezl* with *fez* being intact, whereas *fez*/*fezl* double-mutant mice represent true null conditions. Conversely, in both zebrafish and mouse the over-expression of *fezl*, or *fez* abolishes expression of *shh* in the *zli*. *fezl* over-expression in late gastrula zebrafish embryos expands the prethalamus and hypothalamus territories at the expense of the *zli* and posterior forebrain and/or mid-brain regions. Therefore *fezl*/*fez* gene activity, and specifically *fezf2* activity, plays a key role in formation of the p2/p3 boundary and co-localizes with the *zli* anterior boundary [70,126]. 

By analogy with induction of the midbrain–hindbrain boundary, which develops at the interface of *otx2* and *gbx2* expression, it has been suggested, but not tested, that the interface between the *fezf2* and *irx3* expression domains is the inductive cue at the origin of *zli* formation (reviewed in [3,13,17,44,127]). Indeed, the p2/p3 boundary localizes at the interface between the expression domains of *fezf2* and *irx3,* but also *fezf2* and *otx2,* and *fezf2* and *barhl2* (Figure 1Be and Figure 3). In amphibian, neither over-expression of *irx3,* nor down-regulation of *barhl2,* affects formation of the p2/p3 border [12,32]. Similarly, in zebrafish, the loss of *otx* does not affect *zli* anterior border location [32]. It is possible, however not tested, that Otx2, Barhl2 and Irx TFs have redundant activities in establishment of the interface with the Fezf/Fez TFs that set up the p2/p3 border. Results in frog explants suggest that whereas the p2/p3 limit is important in setting up the *zli* anterior border, it is the co-expression of *barhl2, otx2* and *irx3* that initiates *zli* development. This observation is important, as it indicates that the *zli* developmental mode may be different from that of the midbrain–hindbrain boundary.

Finally, grafts of dorsal diencephalic tissue in chicken inhibit *zli* propagation, arguing that the progression of *shh* expression is limited dorsally by inhibitory factors [38]. The identity of such signals is unknown. However, since *shh* signaling from the *zli* represses *pax6* within the mid-diencephalic furrow [128], Pax6 together with unidentified dorsal signals could in turn prevent *shh* from being expressed beyond the *zli* (Figure 1A,Bf).

In conclusion, the molecular cues initiating *zli* positioning remain partially unknown. In this regard, the determination of signals controlling *otx*, *barhl2* and *irx* neural plate expression will provide important information.

## 10. The GRN Involved in *Zli* Formation is Evolutionarily Conserved

Yao et al. analyzed whether the *cis*- and *trans*-regulatory landscape underlying *zli* development was conserved in the chordate phylum. They investigated conservation of the “*zli* developmental cassette” in the hemichordate *Saccoglossus kowalevskii* (*S. kowalevskii)* that is closest to the central basic reference animal at the root of the chordate phylogenetic tree [129,130]. In *S. kowalevskii*, the narrow band of cells at the proboscis-collar boundary is considered *zli*-like as it expresses *hh*. A study of expression patterns for *barhl2* (*barH*), *otx*, and *irx* orthologs was performed in this hemichordate. Indeed, the proboscis-collar boundary expresses the orthologs of *barhl2*, *otx* and *irx* at the right time and place to perform their patterning function [129,130]. Yao et al. identified in *S. kowalevskii* a 1.1-kb region containing a *cis-*regulatory element containing the six motifs of the mouse SBE1: skSBE1. Functional experiments in mouse and in *S. kowalevskii* demonstrate that skSBE1 is a functional ortholog of the mmSBE1 enhancer [67]. Similar SBE1 *cis-*regulatory elements, intact or with a shuffled motif arrangement, were discovered in lamprey and all jawed vertebrates that display “*zli*-like” structures. In contrast, similar SBE1-like motifs were not found in amphioxus (cephalochordate) or in ascidian (tunicate) that both lack an *hh*-expressing domain in the anterior brain.

In conclusion, these studies support the hypothesis that early chordates inherited an *hh cis-*regulatory-motif from a deuterostome ancestor that was subsequently lost in the invertebrate chordate lineages. A conserved *hh cis-*regulatory-motif (SBE1-like) was maintained in the vertebrate *shh* gene and used to activate its transcription in the *zli*, paving the way for the establishment of this brain-signaling center more than 500 millions years ago [67].

## 11. Forebrain Malformations Associated with Defects in the GRN and Signaling Pathways Supporting *Zli* Formation

The GRN controlling *zli* development is also involved in thalamic primordium induction and in thalamic growth, patterning, and organogenesis. Indeed, depletion of *otx* genes impairs forebrain development (reviewed in [57]). In both frog and mouse, targeted-depletion of *barhl2* generates thalamic developmental defects [12,71]. In *barhl2*^−/−^ mice, p2 thalamic progenitors acquire a pretectal fate and there is an absence of thalamo-cortical axon projections [71]. Finally, *irx* gene depletion in frog, chicken, and zebrafish disrupts thalamus development [3,12,16,17,46,109,131]. Some genetic disorders such as holoprosencephaly (HPE) or the ciliopathies, which are characterized or accompanied by brain malformations in humans and model organisms, are associated with thalamic and thalamo-cortical developmental defects (reviewed in [132,133]). Neuropsychiatric disorders such as obsessive-compulsive disorder and attention deficit hyperactivity disorder have been associated with abnormal neuronal activity within the thalamus (reviewed in [131,134,135,136,137]).

The most prominent pathological condition in which development of the thalamus is affected is HPE. HPE is a rare developmental disorder with an occurrence of 1 case in about 16,000 live births. However, approximately 1/250 conceptuses are thought to be affected by HPE which makes it the most common forebrain defect in humans. HPE presents itself as variable degrees of fusion between the left and right halves of the cerebral hemispheres, basal ganglia and, interestingly, also the thalamus [132,138]. However, rather than representing a fusion event, HPE arises from a failure to separate the two halves of the forebrain along the midline. Most studies focus on correct separation between the cerebral hemispheres (reviewed in [139]). However, little is known on the thalamic contribution to the etiology of HPE.

Mutations in genes linked to HPE have been identified in animal models of HPE and human patients. All of them play important roles in major brain developmental signaling pathways [140,141,142,143,144,145]. Whereas defects in the Shh pathway are the most frequent cause of HPE, genetic screening of HPE patients and studies in animal models also involved the Fgf, Nodal and Notch pathways as major contributors to HPE etiology [140,141,142,143,144,145]. Most mutations found in patients with HPE are in genes that participate in midline formation and early neural plate patterning, strengthening the hypothesis that developmental defects at the origin of these malformations take place early during neural development.

As described above, the Shh pathway relies on primary cilia for signal transduction. It is thus not surprising that mutations in certain cilia-related genes affect diencephalon morphology and induce HPE or HPE-like phenotypes. In mouse, mutants carrying deletions in the anterograde intraflagellar transport (IFT) 172 gene, Shh signaling in the forebrain is down-regulated. Consequently, the mutant diencephalon is severely reduced and embryos exhibit lobar or semilobar HPE [146]. The hypomorphic *cobblestone* allele of another anterograde IFT gene, IFT88, also affects diencephalon development. In this mouse mutant, the border between tel- and diencephalon is dissolved and cells mix freely between the two parts [99], a feature reminiscent of a failure to establish *zli*-mediated compartmentalization and therefore correct establishment of segregation properties. Loss of function of *forkhead box J1 (foxj1)*, a TF regulating the biogenesis of motile cilia, caused striking forebrain defects in *X. laevis* [104]. While cilia on *zli* cells in *Xenopus* are longer than other primary cilia in the wildtype ([104]; Figure 2), *zli* cilia are shortened in *foxj1* morphants and diencephalon size is massively reduced. Taken together, cilia dysfunction in the forebrain leads to variable molecular and morphological defects, pinpointing the dependence of diencephalon development on cilia-based Shh signaling.

In conclusion, even though thalamic fusion in human patients has not yet been connected to diencephalic primordium and *zli* development, it appears possible that malformation of p2 and concurrent mis-patterning may prevent separation of the thalamic complex and plays an as yet unrecognized role in the etiology of HPE. Specifically, failure to establish the anterior medial source of Shh, a lack of competence in the presumptive *zli* region, defective spreading and/or signaling of Shh can cause problems with setting up the p2/p3 border, the *zli*, and correct Shh, Notch and Fgf signaling in the thalamic field. All these events can lead to brain malformations.

## 12. Conclusions

The morphogenetic events supporting brain development are controlled and coordinated by a handful of extracellular signaling networks. Shh is one of the most prominent and collaborates with the Wnt, Bone Morphogenetic Protein, Fgf, Nodal, and retinoid acid (RA) pathways in neurogenesis. The patterning and growth of the diencephalon is, in that sense, particularly meaningful as Wnt, Shh, Fgf, and RA signaling act together in a strictly regulated chronology and topology to orchestrate the development and neurogenesis of the diencephalon and specifically of the thalamus (reviewed in [3,13,17,127]). Whereas recent efforts have allowed partial identification of the *zli* developmental *cis*- and *trans*-determinants, important questions remain unresolved. Specifically, it remains to identify: (i) the molecular cues initiating *zli* emergence and positioning; (ii) the molecular cues driving segregation of *zli* and thalamic cells; and (iii) the contribution of *otx*, *barhl,* and *irx* genes to thalamic development. It will also be interesting to investigate: (i) how the Shh signal is distributed and read in the developing *zli* and the thalamus; and (ii) which signals generate the splitting of the midline in the forebrain region, knowing that this process is defective in HPE. In the years to come, an important focus should be to better understand the gene-environment interactions involved in thalamic organogenesis and to develop animal models (chicken, zebrafish, mouse, *Xenopus*) mimicking human thalamic developmental disorders, a strategy that shall provide critical help into possible clinical interventions.
